# ﻿To what extent are ephippia of Mexican Anomopoda (Crustacea, Cladocera) identifiable?

**DOI:** 10.3897/zookeys.1205.115506

**Published:** 2024-06-24

**Authors:** Gerardo Guerrero-Jiménez, Frida S. Álvarez-Solis, Elaine Aguilar-Nazare, Araceli Adabache-Ortiz, Aleksandra Baquero-Mariaca, Robert L. Wallace, Marcelo Silva-Briano

**Affiliations:** 1 Universidad Autónoma de Aguascalientes, Centro de Ciencias Básicas, Departamento de Biología. Avenida Universidad 940, C.P. 20131, Aguascalientes, Ags. Mexico Universidad Autónoma de Aguascalientes Aguascalientes Mexico; 2 Department of Biology, Ripon College, Ripon, WI 54971, USA Ripon College Ripon United States of America

**Keywords:** Diapausing embryos, dormancy, ephippia, sediment, SEM, taxonomy, ultrastructure

## Abstract

Diapausing embryos encased within cladoceran ephippia result from sexual reproduction and increase genetic diversity. They are also important means by which species bypass harsh environmental conditions and disperse in space and time. Once released, ephippia usually sink to the benthos and remain there until hatching. Using the Sars’ method (incubating sediments to identify cladoceran hatchlings), ephippial egg bank biodiversity can be evaluated. Yet, even when samples are incubated under a variety of conditions, it is not possible to warrant that all have hatched. Few keys are available that facilitate the identification of cladocerans by using only ephippial morphology. Our goal was to analyze some cladoceran ephippia from Mexico, to develop a means to identify them using easily recognizable characteristics. Ephippia of 23 cladoceran species from waters in Aguascalientes (México) in 11 genera (*Alona*, *Biapertura*, *Ceriodaphnia*, *Chydorus*, *Daphnia*, *Dunhevedia*, *Ilyocryptus*, *Macrothrix*, *Moina*, *Pleuroxus*, and *Simocephalus*) were analyzed. In our analysis six morphological features were selected that permitted the identification of ephippia to species(-group) level. The results demonstrate that with a proper catalog of features, some ephippia can be identified.

## ﻿Introduction

Many aquatic micrometazoans produce diapausing embryos (DEs) that permit them to bypass adverse conditions in their habitat, including drought (Schröder et al. 2007), inadequate food conditions (Drillet et al. 2011), and the presence of predators (Hairston and Munns 1984; Slusarczyk 1995; Pijanowska and Stolpe 1996). Taxa capable of producing DEs or other dormant stages include protists (Perrigo et al. 2012), bryozoans (Figuerola et al. 2003), cladocerans (Kokkinn and Williams 1987), copepods (Uye 1985), gastrotrichs (Ricci and Balsamo 2000), nematodes (Ptatscheck and Traunspurger 2020), rotifers (Walsh et al. 2017), and tardigrades (Altiero et al. 2009). The resulting dormant stages in Cladocera usually sink to the bottom, although in some macrothricids, ephippia may be attached to macrophyte leaves (Fryer 1972) or algae (see appendix in Guerrero-Jiménez et al. 2020). Thus, DEs constitute a genetically diverse egg-bank for future generations and they may disperse passively by anemochory (Rivas Jr et al. 2018, 2019), anthropochory (Bailey et al. 2003; Panov et al. 2004; Perrigo et al. 2012), hydrochory (Ricci and Balsamo 2000), and zoochory (Moreno et al. 2019; Vanschoenwinkel et al. 2011). When conditions are favorable, these dormant stages may hatch and replenish the previous population or establish a new one elsewhere (Montero-Pau et al. 2017; Vargas et al. 2019; Odriozola et al. 2020; García-Zamorano and Jiménez-Contreras 2023).

Water fleas (Crustacea, Cladocera) are important components of freshwater ecosystems, passing energy and nutrients on to higher trophic levels (Gulati and DeMott 1998). Most cladocerans reproduce by cyclic parthenogenesis with gamogenetic reproduction happening occasionally. Gamogenetic females of the order Anomopoda Sars, 1865 produce diapausing embryos (resting eggs) covered by a protective, saddle-shaped structure called the ephippium (G., *epi*, on + *hippos*, horse), which usually contains one or two embryos (Fryer 1972); in some macrothricids more than two are observed (Scourfield 1902; Smyly 1956), as well as in Eurycercidae and some other lineages. Some taxa may reproduce dormant stages asexually (Decaestecker et al. 2009), such as an entire population of *Daphniapulex* Leydig, 1860 from North America (Hebert and Crease 1983). Knowledge of the conditions that permit hatching of DEs from sediment samples allows researchers to incubate sediments and identify the hatchlings; from such studies one can develop species lists of the cladocerans in a habitat (Vandekerkhove et al. 2005b). This method is called “the Sars’ Method” (Van Damme and Dumont 2010). Nevertheless, one can never be certain that the DEs from all species present in a sediment sample have hatched, even when the samples have been incubated under a variety of settings that otherwise appear to mimic favorable natural conditions. In addition, hatching is not possible when sediments have been preserved in a fixative. Considering these obstacles, developing comprehensive species lists of a cladoceran community would be improved if researchers were able to identify unhatched ephippia based solely on their morphology. This method is already very widely used in paleolimnology, where cladoceran ephippial identification is one of the pillars of reconstructing past conditions of lakes; entire identification books exist on only remains of cladocerans and ephippia from Europe (Szeroczyfiska and Sarmaja-Korjonen 2007). One of the first advancements in the identification of unhatched ephippia came from drawings of different ornamentations in species of *Bosminalongirostris* complex O.F. Müller, 1785 by Scourfield (1901). Later, Scourfield (1902) expanded on this by illustrating the ephippia of what he called at the time the “Lynceid Entomostraca” (chydorids). Goulden (1966) further improved our knowledge of ephippial morphology by illustrating variation in four *Moina* species. Subsequently, Vanderkerkhove et al. (2004) recognized 29 ephippial morphotypes and Mergeay et al. (2005) observed clear differences in several *Daphnia* species from Kenya. Subfossil records are also a widely used tool for the identification of cladocerans in the field of paleolimnology. In particular, Szeroczyfiska and Sarmaja-Korjonen (2007) provide detailed descriptions of morphological traits of ephippia belonging to several species of Daphniidae and Chydoridae in particular in their ‘Atlas of Subfossil Cladocera from Central and Northern Europe’. More detailed studies were accomplished using SEM technology to illustrate ephippia ornamentations in fossil records, and in ephippia that were still attached to the female (Kotov 2013). Kotov et al. (2018) also demonstrated differences in six *Ceriodaphnia* species from central, northern European Russia, predominantly for their practical use in paleolimnological settings. More recently Guerrero-Jiménez et al. (2020) presented 11 morphotypes extracted from sediments from Spain and Mexico, of different species of water fleas.

Yet, despite the excellent progress that has been made in our ability to differentiate ephippia of different species, we still lack useful diagnostic features. Thus, the aim of our research was to revisit an old – yet important – and largely unstudied question posed more than 30 years ago by Kokkinn and Williams (1987): “is ephippial morphology a useful taxonomic descriptor in the Cladocera?” Studies like Kotov et al. (2018) have shown that it can indeed be useful even between close species, to some extent, yet sometimes large detail is needed. There are at least two major problems to the development of a key to cladoceran ephippia: (1) the information on their taxonomic characters is scattered in the literature and (2) we have insufficient information on their features as a standardized nomenclature of ephippial characters is largely lacking. This knowledge gap is acerbated by the fact that there are at least 620 described cladoceran species (Forró et al. 2007), and most ephippia are studied in Palearctic settings. Thus, we still lack sufficient information so that ephippial morphology can become a useful tool for researchers studying the contemporary ephippial egg bank.

Here we report our results in producing a first database that will hopefully improve our ability to identify cladoceran ephippia of some Mexican taxa based only on their morphology. This information could be an important tool to estimate cladoceran diversity in freshwaters in the region (Vandekerkhove et al. 2005a, b) and subsequently to appreciate their adaptation to the environment within an evolutionary context (Brendonck and De Meester 2003; Gueriau et al. 2016).

## ﻿Materials and methods

### ﻿Study sites and species examined

Sixteen water ponds were analyzed from Aguascalientes, México: 1. El Niagara 2. Tanque de los Jiménez 3. El Tepetate de Abajo, Mal Paso 4. Sierra Fría, Bordo 1: 5. Sierra Fría, Bordo 4; 6. Sierra Fría, Bordo 5: 7. Boca de Túnel; 8. Bordo Siglo XXI; 9. Tapias Viejas 1; 10. El Cedazo Park: 11. Rodolfo Landeros Park: 12. Pulgas Pandas; 13. UAA; 14. Los Gavilanes: 15. El Ocote: 16. Villa Hidalgo. We provide additional details of the study sites in Suppl. material 1: appendix A. We examined ephippia of 23 taxa (Table 1).

**Table 1.** List of the cladoceran taxa examined in this study.

1	*Alonaaguascalientensis* Sinev & Silva-Briano, 2012
2	*Alona* sp.
3	*Biaperturaossiani* Leydig, 1860
4	*Ceriodaphniacornuta* Sars, 1886
5	*Ceriodaphniadubia* Richard, 1894
6	*Ceriodaphnialaticaudata* P.E. Müller, 1867
7	*Ceriodaphniareticulata* Jurine, 1820
8	*Chydorussphaericus* s.l. O.F. Müller, 1776
9	Daphnia (Ctenodaphnia) exilis Herrick, 1895
10	*Daphnialeavis* Birge, 1879
11	*Daphniaparvula* Fordyce, 1901
12	*Daphniapulex* Leydig, 1860
13	*Dunhevediacrassa* King, 1853
14	*Ilyocryptusagilis* Kurz, 1878
15	*Macrothrixmexicanus* Ciros-Pérez, Silva-Briano & Elías-Gutiérrez, 1996
16	*Macrothrixrosea* (Jurine, 1820) – *M.triserialis* Brady, 1886 (see Dumont et al. 2002)
17	*Macrothrixsmirnovi* Ciros-Pérez & Elías-Gutiérrez, 1997
18	*Moinamacrocopa* Straus, 1820
19	*Moinamicrura* Kurz, 1875
20	*Picripleuroxusdenticulatus* Birge, 1879
21	*Simocephalusmixtus* Sars, 1903
22	*Simocephalusvetulus* O.F. Müller, 1776
23	*Simocephalus* sp.

### ﻿Diapausing eggs collection

Using an acrylic tube (2 m × 7.5 cm), we randomly collected sediment samples (cores) from three different points at each study site. We used only the upper 3 cm of cores to extract potentially vital ephippia (Pérez-Martínez et al. 2013). Samples were stored in the dark in a plastic bag at 4 °C. To extract ephippia from sediments we used the sugar flotation method of Onbé (1978) modiﬁed by Marcus (1990). For some species, ephippia were collected from the littoral zone and in other cases from algal mats. Additionally, ephippia of *Simocephalusmixtus* (Sars, 1903) were obtained from a laboratory culture (see details in Suppl. material 1: appendix B).

### ﻿Organism identification and documentation

We recorded a photomicrograph of the ephippia using a Nikon Eclipse light microscope (LM) with a digital camera DS-Fi2 under 4×, 10×, or 20× magnification. To initiate hatching, individual ephippia were placed in wells of a 96-well, polyethylene microplate (CELLTREAT® Scientific Products, 20 Mill St Ste 130, Pepperell, MA 01463) and incubated in a bioclimatic chamber in commercial water (Ciel®, Coca Cola®) under the conditions of 16:8 light/dark period, 20 °C temperature, and white light with an intensity of 345.50 ± 20.54 µmol s^-1^ m^-2^). Once hatched, the females were cultured until they matured, at which time they were identified using the key of Elías-Gutiérrez et al. (2008).

### ﻿SEM analysis

To analyze their ornamentation, ephippia were isolated and fixed in 4% formalin. For SEM study, specimens were dehydrated using a graded ethanol series (60, 70, 80, 90, 96%), after which a critical point drying was performed. Ephippia were attached to a SEM stub (1 cm high × 1.2 cm in diameter) and sputter coated with gold. All samples were observed under a SEM JEOL 5900 LV®, photomicrographs were taken to document ephippial characteristics.

**Figure 1.**
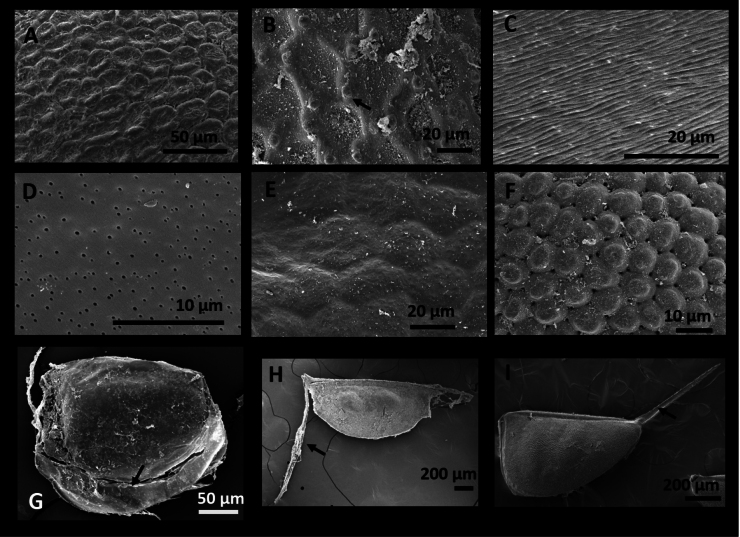
Micrographs of the ornamentation and accessory structure within species studied taken by SEM **A**Depressions (“craters”) in *Moinamacrocopa* Straus, 1820 **B**Verrucae in *Macrothrixmexicanus* Ciros-Pérez, Silva-Briano & Elías-Gutiérrez, 1996, see arrow **C** Striae in *Biaperturaossiani* Leydig, 1860 **D** Pores in *Dunhevediacrassa*; King, 1853 **E** Faint hexagonal reticulation in *Pleuroxusdenticulatus* Birge, 1879 **F** “Scales” with depressions in between in *Macrotrixrosea* Jurine, 1820 **G**Filamentous membrane in *Alonaaguascalientensis* Sinev & Silva-Briano, 2012, see arrow **H** Ventral appendices in Daphnia (Ctenodaphnia) exilis Herrick, 1895, see arrow **I**Spinules on the main posterodorsal spine in *Daphniapulex* Leydig, 1860, see arrow.

*Characterization of ephippia*. To identify ephippia to species level we used six characteristics that we could see using LM and SEM: size, shape, color (including transparency), number of resting eggs, presence of ornamentation and/or accessory structure, and type of ornamentation or accessory structures.

1. Size. Ephippia were categorized into three groups: small (< 400 µm), medium (≥ 400–800 µm) and large (> 800 µm).
2. Shape. Ephippia were classified in geometrical categories: triangular, half oval, square, rectangular, and irregular. In determining the shape of ephippia, accessory structures were not considered.
3. Color. We categorized the color of ephippia when observed under LM.
4. Number of diapausing embryos in an ephippium. The number of diapausing embryos observed inside of the chamber of the ephippium were counted.
5. Type of ornamentation. We recorded features observable by SEM on the surface of ephippia; these included depressions (“craters”), verrucae, striae (“grooves”), pores, reticulations, and scales. “None” indicates the absence of ornamentation and/or accessory structure. **Craters/depressions** defined as small concave holes on the surface of the ephippia with oval or irregular shape present on the surface were categorized as craters (see Fig. 1A). **Verrucae** are identified as small, spherical structures present on some parts of the surface of ephippia as verrucae (see Fig. 1B). **Striae** are pronounced marks in the surface of the ephippia visible by LM (see Fig. 1C). **Pores** have different sizes, but sometimes they were too small to be observed using only LM; in those cases, high resolution SEM was needed (see Fig. 1D). **Reticulations** are smooth marks in the surface of the ephippium with specific patterns that included lines, hexagonal, oval, or irregular shape. Usually, we were not able to observe these by LM; in that case we used the SEM (see Fig. 1E). **Scales** are small, membrane-like structures on the surface of ephippia as scales. These resembled laminar structures, usually covering the entire surface of the ephippium, they could be oval, rounded, or irregular in shape (see Fig. 1F). **Filamentous membranes** are remnants of the membrane that attached it to the ventral portion of the female carapace (see Fig. 1G).
6. Accessory structures. Accessory features were considered to be any extra structures in the basic morphology of the ephippia; these included membranes, peduncles, and spinules. “None” is used when they are absent. Ventral appendices are peduncle-like structure defined as the thin margin attached to the posterior ventral portion of the ephippium (see Fig. 1H). Spinules are labeled as pointed structures present at the posterior portion of ephippia. These varied in size with some being small lying in the margin of the egg (see Fig. 1I).

## ﻿Results

**Figure 2.**
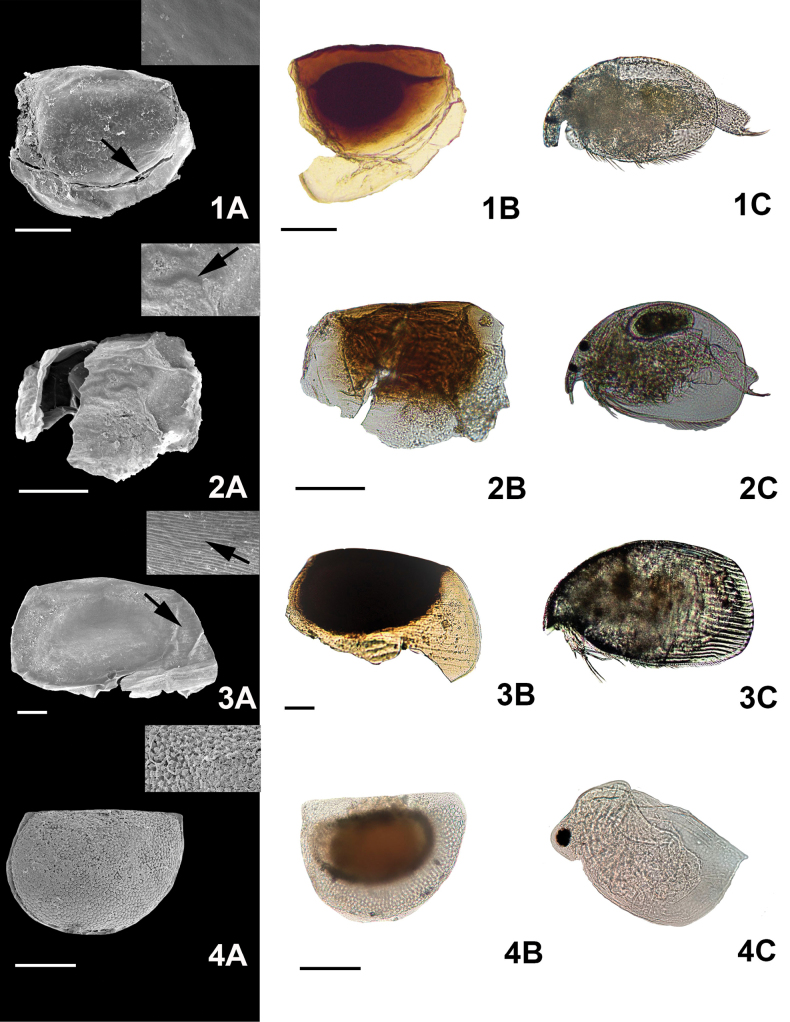
Micrographs of the ephippial ornamentation taken by **A** SEM **B** light microscope, and **C** the organisms hatched from the dormant embryos. 1. *A.aguascalientensis* Sinev & Silva-Briano, 2012 (immature individual in 1C), 2. *Alona* sp., 3. *Biaperturaossiani* Leydig, 1860, 4. *Ceriodaphniacornuta* Sars, 1886. Arrows show the zoom of the specific ornamentation. Scale bars: 100 µm.

**Figure 3.**
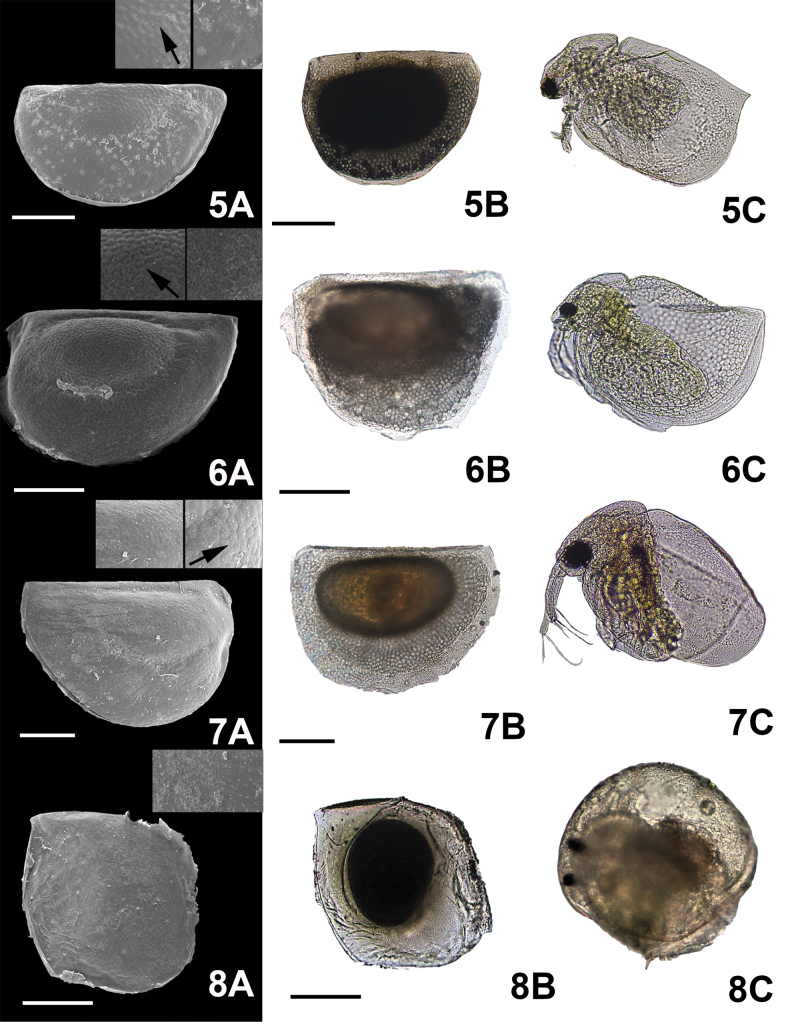
Micrographs of the ephippial ornamentation taken by SEM (**A**) light microscope (**B**) and organism hatched from the egg (**C**). 5. *C.dubia* Richard, 1894, 6. *C.laticaudata* P.E. Müller, 1867, 7. *C.reticulata* Jurine, 1820, 8. *Chydorussphaericus* complex O.F. Müller, 1776. Arrows show the zoomed in image of the ornamentation. Scale bars: 100 µm.

**Figure 4.**
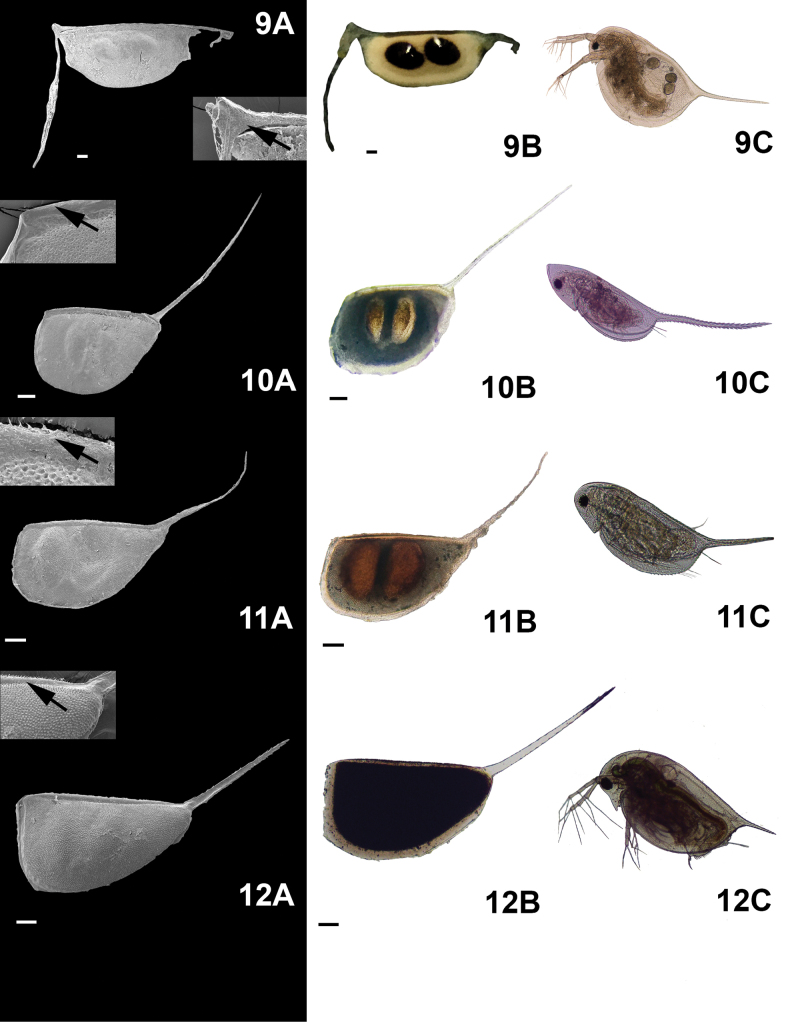
Micrographs of the ephippial ornamentation of some *Daphnia* taxa, taken by SEM (**A**) and light microscope (**B**) and organism hatched from the dormant embryo (some immature) (**C**). 9. Daphnia (Ctenodaphnia) exilis Herrick, 1895, 10. *D.leavis* Birge, 1879, 11. *D.parvula* Fordyce, 1901, 12. *D.pulex* Leydig, 1860. Arrows show the zoomed in image of the ornamentation and some spinules or serrations. Scale bars: 100 µm.

**Figure 5.**
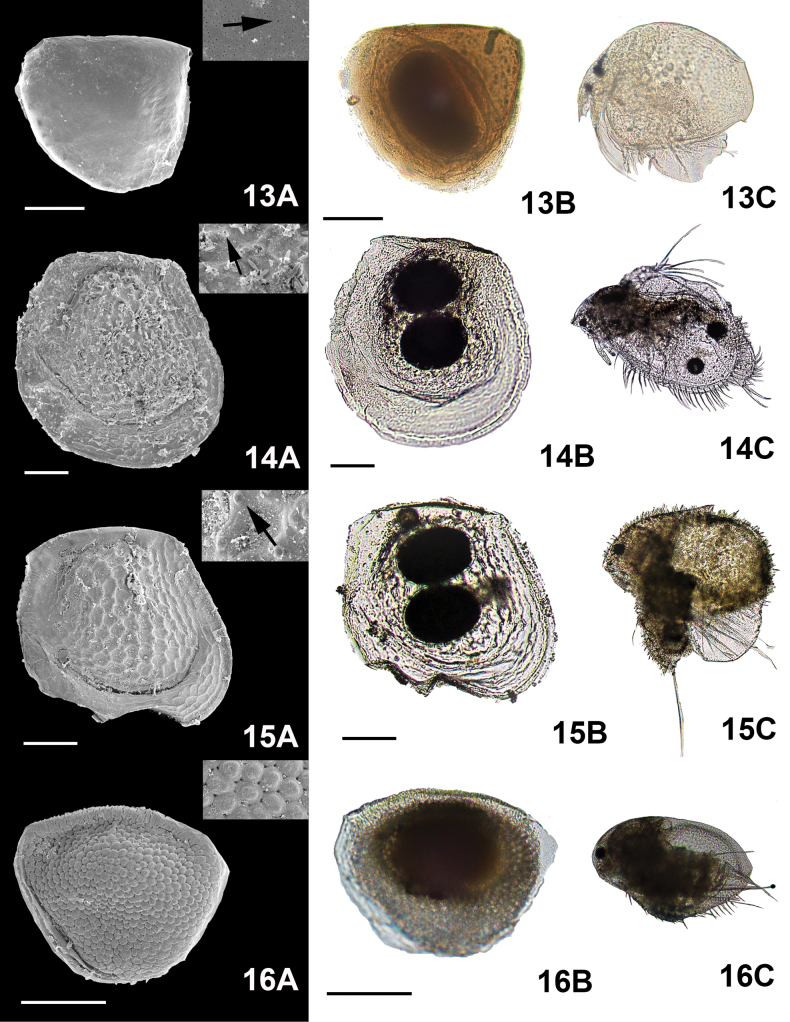
Micrographs of the ephippial ornamentation taken by SEM (**A**) light microscope (**B**) and organism hatched from the egg (some immature) (**C**). 13. *Dunhevediacrassa* King, 1853, 14. *Ilyocryptusagilis* Kurz, 1878, 15. *Macrothrixmexicanus* Ciros-Pérez, Silva-Briano & Elías-Gutiérrez, 1996, 16. *M.rosea* Jurine, 1820. Arrows show the zoomed in image of the ornamentation. Scale bars: 100 µm.

**Figure 6.**
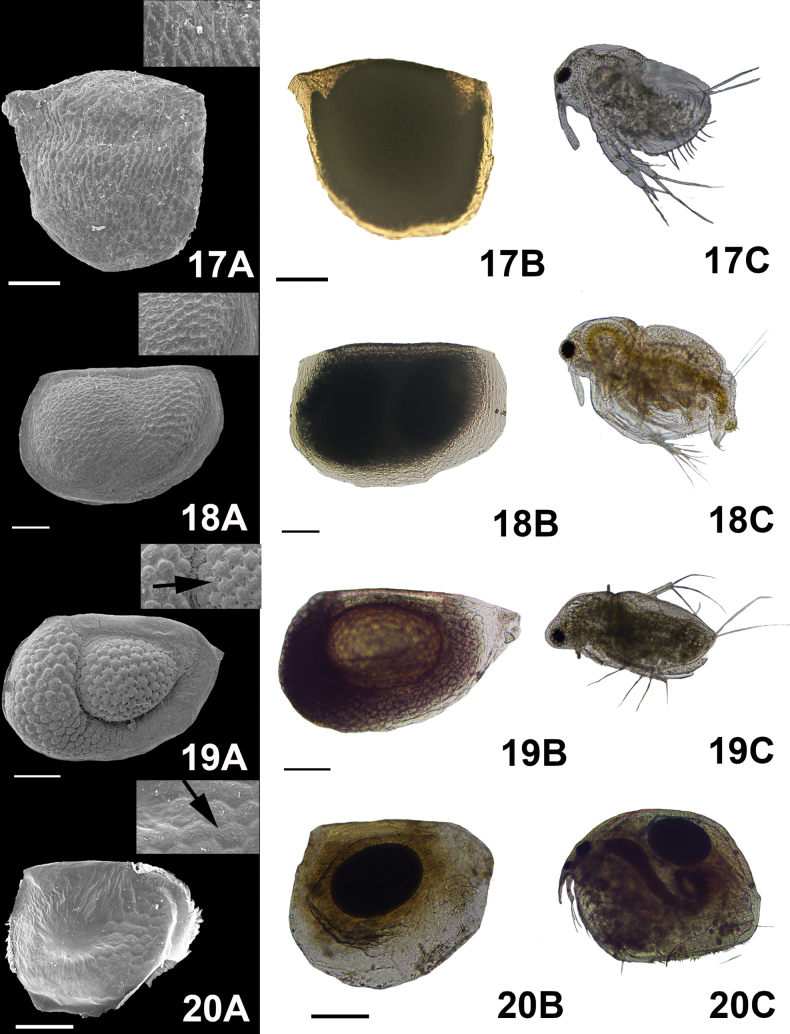
Micrographs of the ephippial ornamentation taken by SEM (**A**) light microscope (**B**) and organism hatched from the egg (**C**). 17. *Macrothrixsmirnovi* Ciros-Pérez & Elías-Gutiérrez, 1997, 18. *M.macrocopa* Straus, 1820, 19. *M.micrura* Kurz, 1875, 20. *Pleuroxusdenticulatus* Birge, 1879. Arrows show the zoomed in image of the ornamentation. Scale bars: 100 µm.

**Figure 7.**
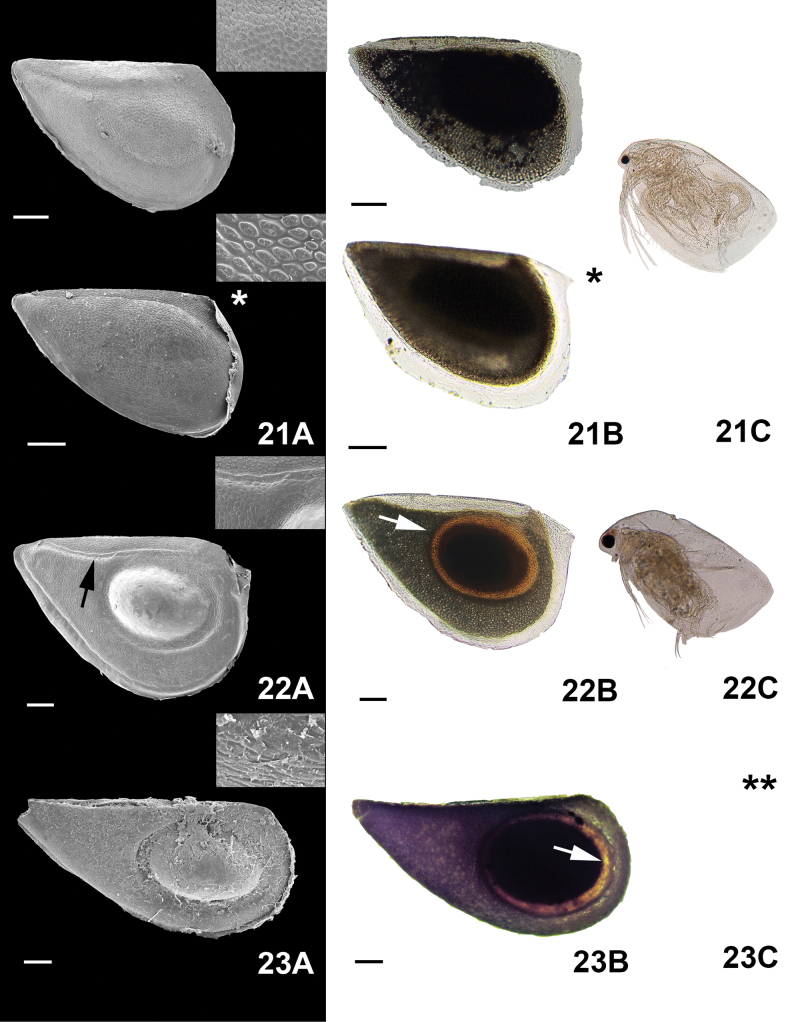
Micrographs of the ephippial ornamentation of *Simocephalus*, taken by SEM (**A**) light microscope (**B**) and organism hatched from the egg (**C**). 21. *Simocephalusmixtus* Sars, 1903, 22. *Simocephalusvetulus* O.F. Müller, 1776, 23. *Simocephalus* sp. (embryo/adult absent). Scale bars: 100 µm. (*) Ephippium extracted from laboratory cultures without males being present; (**) no hatched organism.

A total of 4017 ephippia belong to 23 cladoceran species in 11 genera: *Alona* Baird, 1843, *Biapertura* Smirnov, 1971, *Ceriodaphnia* Dana, 1853, *Chydorus* Leach, 1816, *Daphnia* O.F. Müller, 1785, *Dunhevedia* King, 1853, *Ilyocryptus* G.O. Sars, 1861, *Macrothrix* Baird, 1843, *Moina* Baird, 1850, *Pleuroxus* Baird, 1843, and *Simocephalus* Schoedler, 1858 were analyzed (see Figs 2–7, Suppl. materal 1: appendix B).

1. Size. Within each genus, most species had ephippia of similar size; therefore, while size was useful in identification of the genus, it could not be used for species-level diagnosis (Table 1).
2. Shape. The shape of cladoceran ephippia varied among the genera we examined. Ephippial shape in some genera was consistent: all species of *Simocephalus* had a triangular shape (Fig. 7(21, 22, 23)), while a half oval shape was seen in *Ceriodaphnia* (Figs 2(4), 3(5, 6, 7)). On the other hand, ephippial shape varied in different *Macrothrix* species (Figs 5(15, 16), 6(17)). Another important feature was the margin of the ephippium. The margin of ephippia in *Alona* and *Daphnia* were similar in shape; but in some species the margins were rounded, while in others they were sharp. These differences are enough to distinguish between some species within a genus (Figs 2(1, 2, 3), 4(10, 11, 12)).
3. Color. The most common colors seen in the species studied were brown and dark (Gerrish and Cáceres 2003; Vandekerkhove et al. 2004); however, most ephippia were at least partially transparent. Thus, color was just used in cases that present patterns used to identify ephippia to the level of species: e.g., *Simocephalusvetulus* O.F. Müller, 1776 and *Simocephalus* sp. Within the ephippia, some embryos were orange in color (Fig. 7(22, 23)).
4. “Resting egg” (dormant embryo) number. The number of DEs within the ephippia of the species we studied is reported in Table 2.
5. Type of ornamentation. Five types of ornamentation were observed. (i) ***Depressions* (“*craters*”)** : These were commonly observed in *Daphnia* sp. and *Moinamacrocopa* Straus, 1820. These structures were observed only under SEM (Fig. 6(18A, arrow)). (ii) ***Verrucae***: Small spherical structures were observed only in *Macrothrixmexicanus* Ciros-Pérez et al. 1996 (Fig. 5(15A, arrow)). (iii) ***Striae***: Pronounced marks in the surface of the ephippia were identified; these were linear in *Biaperturaossiani* Leydig, 1860 (Fig. 2(1A, arrow)), smooth and rounded in *Ceriodaphniadubia* Richard, 1894 (Fig. 3(5A, arrow)), pronouncedly rounded in *M.mexicanus* (Fig. 5(15A), and small, interlocking linear in *Simocephalus* sp. (Fig. 7(23A)), no link or patterns between all these species were observed. (iv) ***Pores***: Very small pores were detected on the surface of the ephippia of *Dunhevediacrassa* King, 1853 (Fig. 5(13A, arrow)). (v) ***Reticulation*** : Reticulations were seen in some genera. In *Alona* and *Biapertura* species, linear reticulations were evident (Fig. 2(1, 2, 3)); in *Ceriodaphnia*, the reticulation cells were oval (Figs 2(4), 3(5, 6, 7)); in *Pleuroxus* they were hexagonal in shape (Fig. 6(20A)). (vi) ***Scales***: Different types of scales were observed. Small and irregular ones were present in *Ceriodaphniacornuta* Sars, 1886 (Fig. 2(4A)); rounded scales were seen in the *Macrothrixrosea* Jurine, 1820 (see Dumont et al. 2002) (Fig. 5(16A)); oval scales were present in *S.mixtus* and *S.vetulus* (Fig. 7(21A, 22A)). Finally, in *Moinamicrura* Kurz, 1875, two different types of scales were detected: rounded scales in the anterior portion of the ephippium and scales with irregular shape covering the section where the DE was located (Fig. 3(5A)).
6. Accessory structures. Only five species: *Alonaaguascalientensis* Sinev and Silva-Briano, 2012, Daphnia (Ctenodaphnia) exilis Herrick, 1895, *Daphnialeavis* Birge, 1879, *D.parvula* Fordyce, 1901, and *D.pulex* Leydig, 1860 have accessory structures. Most of those were seen in *Daphnia* (Table 2, Fig. 4). (vii) ***Filamentousmembranes***: Filamentous membranes were observed only in *A.aguascalientensis* (Fig. 3(5A)). (viii) ***Ventral appendices***: These were only observed in *C.exilis* (Fig. 3(5A)). (ix) ***Spinules***: Spinules were specific to *Daphnia*, but they were also observed in *C.exilis*. In *Daphnia*, a long caudal spinule is present (Fig. 3(5A)), while in *C.exilis*, both caudal spinule and anterior projection were observed. In the posterior portion, a small spinule was attached to the ventral appendix while a longer spinule is observed in the anterior part of the ephippium (Fig. 3(5A)).

## ﻿Discussion

### ﻿Taxon-specificity in ephippial morphology

Here we provide data of six morphological characteristics on ephippial morphology on 23 taxa (Figs 2–7). Most of our results provide evidence of taxon-specificity in ephippial morphology among the studied species, but there were several challenges. For example, identification of *Ceriodaphnia* species was problematic. When using light microscopy, Vanderkerkhove et al. (2004) also reported difficulty in identifying *Ceriodaphnialaticaudata* P.E. Müller, 1867, *Ceriodaphniapulchella* (Sars, 1862), *Ceriodaphniaquadrangula* O.F. Müller, 1785, and *Ceriodaphniareticulata* (Jurine, 1820). On the other hand, Kotov et al. (2018) were able to differentiate among four species. Berner (1985) in a study on the *Ceriodaphniacornuta* complex, found high variability between species and proposed the separation of *C.cornuta* and *C.rigaudi* by comparing different details of the ephippia. In SEM studies, we also found that *C.cornuta* differed from other species in the genus. However, the smooth surface of ephippia in *C.dubia*, *C.laticaudata*, and *C.reticulata* made taxonomical distinctions difficult. Another example is seen in ephippia of *S.mixtus*: those from cultures showed clear ornamentation, while those from sediments did not. We also note that in some *Daphnia* species ephippia from sediment samples did not always retain their posterior spinulae. Although differences between some external ornamentations in cladocerans are linked to plasticity, the latter ephippial structure may degrade while in the sediment. Thus, ornamentations or appendages can be present when ephippia are formed in cultures, but may be absent in specimens collected from sediments, as seen in many of our *Daphnia* samples.

**Table 2.** Taxonomic standardization with six categories to identify ephippia in 11 genera of cladocerans.

Genus	Egg size (µm)	Egg shape	Color	RE	Type of ornamentation	Accessory structure	Species
*Alona*	Small (< 400)	Rectangular	Brown and dark in the resting egg chamber	1	None	Width filamentous membrane in the base of the egg	*A.aguascalientensis*
		Rectangular	Light brown	1	Striae	None	*A.* sp.
*Biapertura*	Medium (≥ 400–800)	Rectangular	Black and transparent membrane	1	Thin linear reticulations and soft parallel striae	None	*B.ossiani*
*Ceriodaphnia*	Small (< 400)	Semi-circular	Transparent and brown in the resting egg chamber	1	Irregular and small scales in all egg	None	*C.cornuta*
	Small (< 400)	Semi-circular	Transparent and dark in the resting egg chamber	1	Soft rounded striae in the resting egg portion	None	*C.dubia*
	Small (< 400)	Semi-circle	Transparent and brow in the resting egg chamber	1	Very soft oval reticulations in the margin of the egg but more visible in the resting egg portion	None	*C.laticaudata*
	Small (< 400)	Semi-circle	Transparent and brown in the resting egg chamber	1	Very soft oval reticulations	None	*C.reticulata*
*Chydorus*	Small (< 400)	Square	Light brown and dark in the resting egg chamber	1	None	None	*C.sphaericus*
*Daphnia*	Large (> 800)	Rectangular	White and dark in the resting egg chamber	2	Small irregular reticulations	Spinule and ventral appendix	D. (Ctenodaphnia) exilis
	Large (> 800)	Rectangular	Dark and surrounded by transparent membrane	2	small craters	Large and thin spinule	*D.leavis*
	Medium (≥ 400–800)	Triangular	Transparent grey and dark in the resting egg chamber	2	small craters	Large and thin spinule, but wider in the base	*D.parvula*
	Medium (≥ 400–800)	Triangular	Dark and surrounded by transparent membrane	2	small craters	Large spinule and width	*D.pulex*
*Dunhevedia*	Small (<400)	Half oval	Brown and dark brown in the resting egg chamber	1	Several small pores	None	*D.crassa*
*Ilyocryptus*	Medium (≥ 400–800)	Oval	Transparent	2	Oval striae with apical small verrucae	None	*I.agilis*
*Macrothrix*	Medium (≥ 400–800)	Irregular	Transparent and dark resting eggs	2	Oval striae with apical verrucae	None	*M.mexicanus*
	Small (< 400)	Half oval	Transparent brown and Dark in the resting egg chamber	1	Rounded scales	None	*M.rosea*
		Square	Dark and surrounded by transparent membrane	2	None	None	*M.smirnovi*
*Moina*	Medium (≥ 400–800)	Rectangular	Dark rounded by transparent membrane	2	Craters	None	*M.macrocopa*
	Medium (≥ 400–800)	Oval	Brownish orange and dark in the resting egg chamber	1	Rounded and irregular scales	None	*M.micrura*
*Pleuroxus*	Medium (≥ 400–800)	Rectangular	Light brown and resting eggs dark	1	Hexagonal reticulation in the posterior portion	None	*P.denticulatus*
*Simocephalus*	Medium (≥ 400–800)	Triangular	Dark surrounded by transparent membrane	1	Oval scales	None	*S.mixtus*
	Large (> 800)	Triangular	Different gray tonalities and the resting egg chamber orange	1	Scales and a margin that round the egg	None	*S.vetulus*
	Large (> 800)	Triangular	Dark surrounded by transparent membrane and resting egg chamber orange	1	Interlocking linear small striae	None	*S.* sp.

**RE** = resting egg

Does form follow function (Gould 1971) in cladoceran ephippia or is their morphology simply the residue of their formation while part of the female? This is a difficult question to answer. For example, ornamentation in the ephippium of *Chydorussphaericus* complex is the same as the shell ornamentation in the female adult (see Fig. 2(8A–8C)). Despite the difficulty in coming to specific conclusions about the diversity of *Chydorussphaericus* group (Kotov et al. 2016), ephippial eggs were still useful to identify the species group. In *Daphnia* species, spinules in the resting eggs are also present in the female (see Fig. 3(9–12A, B, C)). On the other hand, *Moinamicrura*, *Ilyocryptusagilis*, *Macrothrixmexicanus*, and *Simocephalusvetulus* are clear examples of different ornamentations in diapausing eggs in comparison to the female. These examples could suggest that these structures possess a specific function during dormancy. However, we must remember that selection pressures do not operate on a single trait, they work on the entire animal within its environment: this is referred to as the Pareto optimality (Tendler et al. 2015). Thus, any research on ephippia must consider the importance of ephippial characteristics in the ephippial female, as well as while it resides in the sediment. For instance, the peduncle presents in *Daphniapulicaria* Forbes, 1893 from Sierra Nevada (Spain) might be used for dispersion or to remain in the surface of the lake, as was observed by Guerrero-Jiménez et al. (2020). In *S.vetulus*, the margin of the ephippia is ornamented and the chamber in which the DE resides is orange eggs were mostly floating (see Suppl. material 1: appendix B). Different levels of carotenoids produce the orange pigmentation present in ephippia of cladoceran species permitting greater light absorption, which could improve hatching (Stross, 1966). On the other hand, spinules on ephippia of *Daphnia* spp. are probably a residual of the structures that protect adult females from predators.

In Mexico, approximately 150 cladoceran species have been reported (Elías-Gutiérrez et al. 2008), so our study has only covered a small proportion (15%) of the total diversity. This number is likely to increase simply because only 1% of watersheds in the area have been explored (Elías-Gutiérrez et al. (1999). While our work analyzed 23 morphotypes this represents the beginning of the studies in ephippia structures from subtropical areas. We urge that more work should be done on this subject, including paleolimnological research, such as an adaptation to a (sub)tropical context of the ‘Atlas of Subfossil Cladocera from Central and Northern Europe’ by Szeroczyfiska and Sarmaja-Korjonen (2007). Additionally, by combining light microscopy and SEM techniques, useful results could be obtained. An example of this is the remarkable work of Kotov (2013), who demonstrated that even in subfossil samples, good ultrastructure of the ornamentation could be obtained, leading to positive identifications at detailed levels.

In this work, we could identify some useful diagnostic traits for different morphotypes, even for some where high plasticity is well known, such as the *Chydorussphaericus* complex, *Daphnia*, and *Alona*. However, we keep in mind that this condition will surely change when research of species increases. We recommend studying the ornamentations of ephippial structures to detect the boundaries for each taxon or complex of species, and ultimately, contribute to understand the evolution and biodiversity of water fleas in contemporary and past lakes. Our efforts augment current information which could shed light on cryptic speciation in cladocerans as several complex groups have been reported by Petrusek et al. (2003), Elías-Gutiérrez and Valdez-Moreno (2008), Quiroz-Vázquez and Elías-Gutiérrez (2009), and Bekker et al. (2016). While identification of cladoceran ephippia by itself has proven difficult (Murat and Sevil 2014), improving our ability to identify them to species level, will extend our knowledge of cladoceran distribution, especially to habitats that cannot be sampled throughout the year: i.e., those that are too remote and ephemeral ponds.

## ﻿Conclusions

We found that ephippial morphology was relatively consistent and contained useful diagnostic features. Our results show useful differences in the morphology of ephippia in the taxa encountered, except in *Ceriodaphnia*; thus, morphological differences of ephippia in that genus remain a challenge. Because the six features we used in this study allowed us to achieve a useful identification of the ephippia we examined, we conclude that morphological characterization of ephippia is a sufficiently robust tool for the identification of ephippia, for certain taxa. Nevertheless, we recognize that a serious knowledge gap remains. Our analysis and the database we provide needs to be expanded to include many more species and additional stable characteristics. When this is achieved, ephippial morphology will be a convenient and practical means for cladoceran morphological identification.

## ﻿Additional information

### Author ORCIDs

Gerardo Guerrero-Jiménez https://orcid.org/0000-0002-1788-8099

Frida S. Álvarez-Solís https://orcid.org/0009-0003-2188-0078

Elaine Aguilar-Nazare https://orcid.org/0009-0002-7090-6038

Araceli Adabache-Ortiz https://orcid.org/0000-0003-4332-9650

Aleksandra Baquero-Mariaca https://orcid.org/0009-0005-3765-7414

Robert L. Wallace https://orcid.org/0000-0001-6305-4776

Marcelo Silva-Briano https://orcid.org/0000-0002-5372-2408

### Data availability

All of the data that support the findings of this study are available in the main text or Supplementary Information.

## Supplementary materials

Supplementary material 1 Supplemetary data Data type docx Explanation note map with coordinates of all locations where samples were collected; table with the distribution and the number of the ephippia morphotypes found. In addition, a figure with all morphotypes identified and used for experiments; picture of a small wood piece with several ephippia of *Simocephalusmixtus*. https://binary.pensoft.net/file/1078331This dataset is made available under the Open Database License (http://opendatacommons.org/licenses/odbl/1.0/). The Open Database License (ODbL) is a license agreement intended to allow users to freely share, modify, and use this Dataset while maintaining this same freedom for others, provided that the original source and author(s) are credited.Gerardo Guerrero-Jiménez, Frida S. Álvarez-Solis, Elaine Aguilar-Nazare, Araceli Adabache-Ortiz, Aleksandra Baquero-Mariaca, Robert L. Wallace, Marcelo Silva-Briano
