# Supplementary material for: ﻿To what extent are ephippia of Mexican Anomopoda (Crustacea, Cladocera) identifiable?
Source: Zookeys. 2024 Jun 24;1205:169–89. doi: 10.3897/zookeys.1205.115506 (PMC11217647; doi:10.3897/zookeys.1205.115506)
Supplement: Supplementary material 1 — Supplemetary data [file zookeys-1205-169_article-115506__-s001.docx]

**To what extent are ephippia of Mexican Anomopoda (Crustacea, Cladocera) identifiable?**

GERARDO GUERRERO-JIMÉNEZ, FRIDA S. ÁLVAREZ-SOLIS, ELAINE AGUILAR-NAZARE, ARACELI ADABACHE-ORTIZ, ALEKSANDRA BAQUERO-MARIACA, ROBERT L. WALLACE, & MARCELO SILVA-BRIANO

**Supplementary Information for:**

Three Appendixes:

**Appendix A:** Map with coordinates of all locations where samples were collected.

**Appendix B:** Table with the distribution and the number of the ephippia morphotypes found. In addition, a figure with all morphotypes identified and used for experiments.

**Appendix C:** Picture of a small wood piece with several ephippia of *Simocephalus mixtus.*

**Appendix A.**


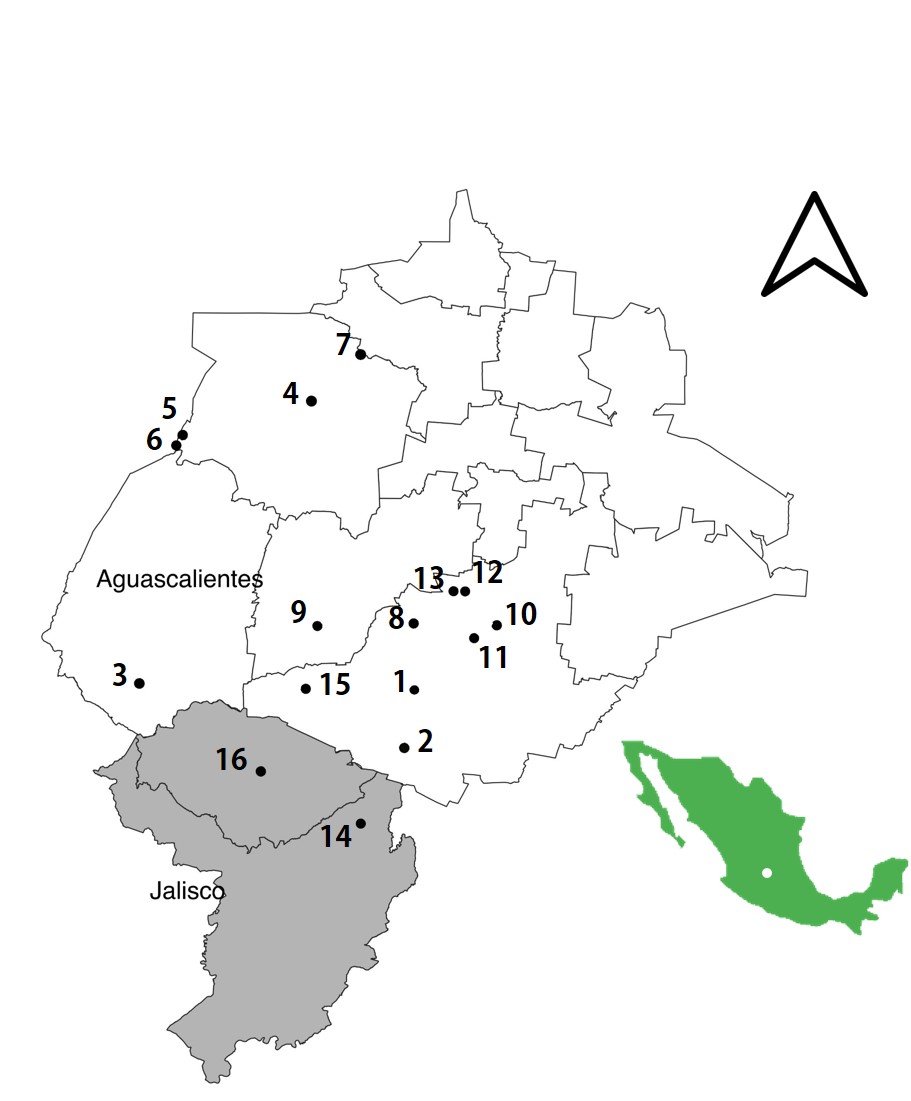


Map with the coordinates where samples were collected. **1.** **El Niagara** 21° 46´47” N, 102° 22´10” W; **2.** **Tanque de los Jiménez:** 21° 42´ 06” N, 102° 22´ 58” W; **3.** **El Tepetate de Abajo, Mal Paso:** 21°47´21” N, 102°44´34” W; **4. Sierra Fría, Bordo 1:** 22°10´20” N, 102° 30´33” W; **5. Sierra Fría, Bordo 4:** 22° 07´34” N, 102° 41´01” W; **6. Sierra Fría, Bordo 5:** 22° 06´44” N, 102° 41´32” W; **7. Boca de Túnel:** 22° 14´ 07” N, 102° 26´32” W; **8. Bordo Siglo XXI:** 21° 52´ 14” N, 102° 22´ 12” W; **9. Tapias Viejas 1:** 21° 52´ 02” N, 102° 30´ 03” W; **10. El Cedazo Park:** 21° 52´05” N, 102° 15´26” W; **11. Rodolfo Landeros Park:** 21° 51´ 03” N, 102° 17´17” W; **12. Pulgas Pandas:** 21° 54´ 51” N, 102° 18´01” W; **13. UAA:** 21° 54´52” N, 102° 18´58” W; **14.** **Los Gavilanes:** 21° 35´ 57” N, 102° 26´ 31” W; **15. El Ocote**: 21° 46´ 55” N, 102° 30´59” W. **16. Villa Hidalgo:** 21° 40´12” N, 102° 34´39” W.

**Appendix B.** Number of ephippia collected for each cladoceran species in every lake sampled *in situ* and from cultures, the total of organisms hatched, and the place where ephippia were found.

| **No.** | **Species** | **Lake/propagules per lake** | | **#**  **of hatched** | | **Place where ephippium were found** |
| --- | --- | --- | --- | --- | --- | --- |
| 1 | *A. aguascalentensis* | Tapias Viejas  Tanque de los Jiménez | 5  6 | 3  2 | Sediment  Sediment | |
| 2 | *Alona* sp. | Sierra Fria, Bordo 1 | 3 | 1 | Sediment | |
| 3 | *Biapertura ossiani* | Sierra Fría, Bordo 4 | 8 | 3 | Sediment | |
| 4 | *Ceriodaphnia cornuta* | Tapias Viejas | 9 | 2 | Sediment | |
| 5 | *C. dubia* | Rodolfo Landeros Park  Siglo XXI | 15  40 | 1  6 | Attached to algae  Attached to algae | |
| 6 | *C. laticaudata* | Ocote | 2 | 1 | Sediment | |
| 7 | *C. reticulata* | El Tepetate de Abajo, Mal Paso. | 14 | 3 | Attached to algae | |
| 8 | *Chydorus sphaericus* | Los Gabilanes | 5 | 2 | Attached to algae | |
| 9 | *Daphnia (Ctenodaphnia) exilis* | Cedazo Park  Rodolfo Landeros Park | 103  9 | 5  1 | Littoral floating  Sediment | |
| 10 | *D. laevis* | Tapias Viejas  Rodolfo Landeros Park  Villa Hidalgo | 7  1  30 | 4  1  2 | Sediment  Sediment  Sediment | |
| 11 | *D. parvula* | Parque Rodolfo Landeros Park  Ocote  Boca de Túnel | 7  4  60 | 3  3  5 | Littoral floating  Sediment  Sediment | |
| 12 | *Daphnia pulex* | El Cedazo Park  Rodolfo Landeros Park | 40  5 | 1  2 | Littoral floating  Sediment | |
| 13 | *Dunhevedia crassa* | Rodolfo Landeros Park  Siglo XXI | 1  3 | 1  0 | Sediment  Sediment | |
| 14 | *Ilyocryptus agilis* | Sierra Fría, Bordo 5 | 4 | 2 | Sediment | |
| 15 | *Macrothrix mexicanus* | Tanque de los Jiménez | 1 | 1 | Sediment | |
| 16 | *M. rosea* | Tapias Viejas  Sierra Fría, Bordo 5 | 30  15 | 4  6 | Sediment Sediment | |
| 17 | *M. smirnovi* | Tanque de los Jiménez | 1 | 1 | Sediment | |
| 18 | *Moina macrocopa* | Pulgas Pandas  El Cedazo Park  UAA  Niagara | 50  500  254  458 | 6  4  50  86 | Sediment  Sediment  Sediment  Sediment | |
| 19 | *M. micrura* | UAA  Niagara | 120  65 | 8  18 | Sediment  Sediment | |
| 20 | *Picripleuroxus denticulatus* | El Tepetate de Abajo, Mal Paso. | 65 | 15 | Attached to algae | |
| 21 | *Simocephalus mixtus* | Boca de Túnel  Rodolfo Landeros Park  Rodolfo Landeros Park Culture | 2000  12  10 | 2  1  4 | Littoral Floating  Sediment  Lying in the bottom | |
| 22 | *S. vetulus* | Tapias Viejas | 54 | 3 | Floating | |
| 23 | *Simocephalus* sp. | Tapias Viejas | 1 | 0 | Sediment | |

**Appendix C.**

**
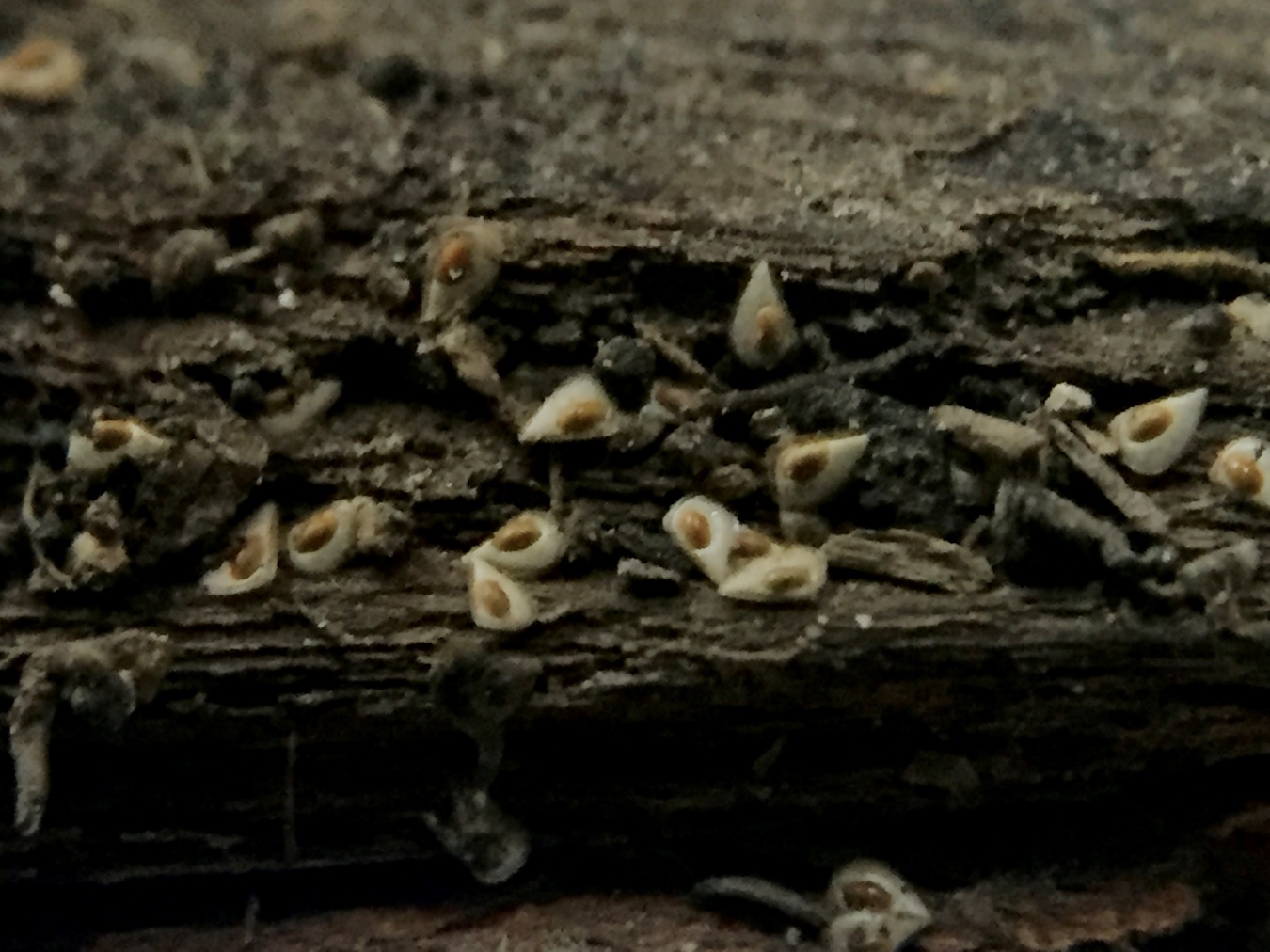
**

Small wood piece with several ephippia of *Simocephalus mixtus,* however, we observed millions of eggs in the littoral section of the water pond.
